# ARE STATINS THAT GOOD? ASSESSING THE PROTECTIVE EFFECT OF STATINS AMONG OLDER ADULTS WITH SERIOUS ILLNESS OR INJURY

**DOI:** 10.1093/geroni/igae098.3791

**Published:** 2024-12-31

**Authors:** Sandra Sanchez-Reilly, Siva Naga Srinivas Yarrarapu, Erin Sidle, Marla Khalikov, Jennifer LaCoss, Elizabeth Reilly, Shannon Crenshaw, Ashley McGinity

**Affiliations:** South Texas Veterans Health Care System and the University of Texas Health Science Center at San Antonio, San Antonio, Texas, United States; UTHSCSA, San Antonio, Texas, United States; University Health, San Antonio, Texas, United States; University Health System, University Health System, Texas, United States; UT Health San Antonio, UT Health San Antonio, Texas, United States; Health Careers High School, Health Careers High School, Texas, United States; University Health, San Antonio, Texas, United States; University of Texas Health San Antonio, San Antonio, Texas, United States

## Abstract

**Background:**

Older Adults (OA) constitute the majority of Emergency Department (ED) visits, largely due to fall-related injuries and acute serious illnesses (ASI). OA’s ED visits lead to potentially extended hospitalizations and increased healthcare costs. Statins, commonly prescribed for cholesterol-lowering effects/cardiovascular benefits, are widely used among OA. Few inconclusive studies have explored the relationship between statins and OA falls/ASI. Objective: To investigate the relationship between statins and its protective effects among OA who visit ED due to injury/ASI.

**Methods:**

OA ED visits (age>65) were collected for seven weeks (summer 2024) at level-1 trauma center. Variables included acuity score (1-4, 1 being worse), diagnosis, ICU admission need, death and statins usage prior to event.

**Results:**

1771 OA-ED. Median age 73 (68-80), 52% female (923), 34% White, 55% Hispanic. Acuity: 1(3%), 2(33%), 3(59%), 4(5%). OA statins usage was 50%. 25% visits were falls, followed by infections (13%), GI complaints (11%) among others. 50% OA went home, 43% admitted. 12% became ICU admissions, 2% died. Among statin-takers, 13.2% were admitted to ICU vs.11.4% not-on-statins (p=0.27,NS). Interestingly, 1.3% statin-takers died because of the injury/ASI that brought them to ED, vs. 2.6% not-on-statins (p=0.04). Falling was the most common reason for seeking ED: 21.6% statin-takers vs. 27.9% not-on-statins (p=0.002), suggesting a protective effect.

**Conclusions:**

Statins appear to have a significant protective effect on falls/ASI among OA, specifically pertaining to decreasing their fall risk and in-hospital mortality rate. Further studies should explore prophylactic ways to use statins in improving hospital-related outcomes and decreasing risk of geriatric syndromes.

